# Real-world comparative effectiveness of ARNI versus ACEi/ARB in HF with reduced or mildly reduced ejection fraction

**DOI:** 10.1007/s00392-022-02124-w

**Published:** 2022-11-29

**Authors:** Michael Fu, Aldina Pivodic, Oskar Käck, Madlaina Costa-Scharplatz, Ulf Dahlström, Lars H. Lund

**Affiliations:** 1 Department of Molecular and Clinical Medicine, Institution of Medicine, University of Gothenburg, Gothenburg, Sweden; 2 Statistiska Konsultgruppen, Gothenburg, Sweden; 3 Department of Clinical Neuroscience, Institute of Neuroscience and Physiology, Sahlgrenska Academy, University of Gothenburg, Gothenburg, Sweden; 4 Novartis Sweden AB, Stockholm, Sweden; 5 Department of Cardiology, Linköping University, Linköping, Sweden; 6 Department of Health, Medicine and Caring Sciences, Linköping University, Linköping, Sweden; 7 Cardiology Unit, Department of Medicine, Karolinska Institutet, Stockholm, Sweden

**Keywords:** Heart failure, Heart failure with reduced ejection fraction, Heart failure with mildly reduced ejection fraction, ARNI, Real-world, Effectiveness

## Abstract

**Aims:**

Sacubitril/valsartan is a first-in-class angiotensin receptor–neprilysin inhibitor (ARNI) with a class-1 guideline recommendation. We assessed the real-world effectiveness of ARNI versus angiotensin-converting enzyme inhibitor/angiotensin receptor blocker (ACEi/ARB) on all-cause and cardiovascular (CV)-related mortality and hospitalizations in heart failure (HF) with reduced or mildly reduced ejection fraction (EF).

**Methods:**

Patient-level clinical, laboratory, drug dispensation, hospitalization, and mortality data were derived from the Swedish Heart Failure Registry (SwedeHF) and interlinked databases (1 April 2016–31 December 2020). Eligible ARNI:ACEi/ARB patients (*n* = 7275:24,604) had a left ventricular EF < 50%. Mortality and hospitalizations with ARNI (≤ 3 months pre-/post-1 April 2016 index [SwedeHF]; *n* = 1506) versus ACEi/ARB (≤ 3 months post-index; *n* = 17,108) were assessed using propensity score matching (1:1 ratio) with clinical variables, and sensitivity analysis (1:2/1:3 with, and 1:2 without clinical variables).

**Results:**

ARNI induced a 23% reduction in all-cause mortality versus ACEi/ARB (1:1 hazard ratio [HR; 95% confidence interval (CI)]: 0.77 [0.63–0.95], *p* = 0.013), and a non-significant 23% relative risk reduction in CV-related mortality (0.77 [0.54–1.09], *p* = 0.13), but no difference in all-cause or CV-related hospitalization (1.02 [0.91–1.13]; *p* = 0.76; 1.01 [0.91–1.15]; *p* = 0.84, respectively). Sensitivity analyses confirmed all-cause mortality was reduced for ARNI versus ACEi/ARB (HR 0.90 [95% CI 0.82–0.99], *p* = 0.026), but not CV-related mortality (HR 1.04 [95% CI 0.89–1.22], *p* = 0.63).

**Conclusions:**

In this nationwide real-world study including a population of patients with HF with reduced or mildly reduced EF, ARNI as part of guideline-led Swedish clinical practice was associated with a statistically significant relative risk reduction in all-cause mortality compared with ACEi/ARB.

**Supplementary Information:**

The online version contains supplementary material available at 10.1007/s00392-022-02124-w.

## Introduction

In the pivotal PARADIGM-HF trial, sacubitril/valsartan as the first-in-class angiotensin receptor–neprilysin inhibitor (ARNI) was associated with a 20% relative reduction in the risk of cardiovascular (CV) death and heart failure (HF)-related hospitalization (primary endpoint) in adult patients with symptomatic chronic HF with reduced ejection fraction (HFrEF; left ventricular ejection fraction [LVEF] ≤ 40%) compared with angiotensin-converting enzyme inhibitor (ACEi) [1]. Furthermore, the phase 3 randomized PARAGON-HF trial that enrolled patients with an LVEF ≥ 45% demonstrated a 13% non-significant reduction in total (first and recurrent) hospitalizations (primary endpoint; *p* = 0.06), and a 22% significant reduction in HF-related hospitalizations and CV-related death with ARNI versus angiotensin 2 receptor blocker (ARB) in a prespecified patient population with LVEF ≤ 57% [2]. ARNI received national reimbursement in Sweden according to the regulatory label in April 2016. In line with the 2016 European Society of Cardiology (ESC) guidelines, patients in Sweden were recommended to have received optimal treatment with ACEi or ARB, and mineralocorticoid receptor antagonists (MRAs) prior to initiating ARNI [3, 4].

Randomized controlled trials remain the gold standard for regulatory approval, and real-world analyses conducted using robust methodology can provide evidence supplementing clinical trial data [5]. Several observational studies and systematic reviews have assessed the clinical effectiveness of ARNI versus ACEi or ARB in the real-world setting [6, 7] and reported a 10–25% reduction in all-cause mortality, a 10–16% reduction in CV death, and a 10–38% reduction in HF hospitalizations with ARNI compared with standard of care (including ACEi/ARB) [6]. However, as is the nature of retrospective studies and pre-existing data, there is often heterogeneity between study cohorts and methodologies among studies. Moreover, inequality among health care systems in different countries impact implementation of new therapies.

The Swedish universal health care system may differ from the cohorts on whom previous analyses were performed regarding the equity of care provided to all patients. For example, Swedish national requirements include previous therapies with highest optimal tolerable doses including beta blocker, ACEi/ARB, MRA, and cardiac resynchronization therapy (CRT) before initiation of ARNI, which may differ from other countries.

To date, there is no published evidence in Europe on the comparative effectiveness of ARNI versus. ACEi on all-cause mortality in a population representative of real-world HF including HFrEF and HF with mildly reduced ejection fraction (HFmrEF). This is despite the emphasis in the 2021 ESC guidelines that patients with HFmrEF have some degree of LVEF reduction, and therefore may benefit from similar therapies to those with HFrEF including ARNI [8]. Sweden has several comprehensive nationwide data sets that facilitate the identification of clinical outcomes in patients treated for HF [3, 9, 10]. Given the granularity and coverage of these databases, registry-based findings in Sweden provide one of the world’s most reliable real-world observations with the most comprehensive outcome data from clinical practice worldwide [11]. Hence, providing relevant information not only for the Nordics, but also for the broader international HF community.

This study utilized patient-level data from the Swedish Heart Failure Registry (SwedeHF), linked to clinical outcomes derived from three National Administrative Health Registries, to assess the comparative effectiveness of ARNI versus ACEi/ARB in patients with an LVEF < 50% in Sweden, using robust matching methodology to account for group differences and adjust for confounding factors.

## Methods

### Study design and settings

A retrospective comparative effectiveness non-interventional cohort analysis was performed using propensity score matching to compare real-world mortality and hospitalization in patients with HFrEF or HFmrEF (defined as LVEF < 40% and 40–49%, respectively) in Sweden who received either guideline-directed ARNI or ACEi/ARB along with other guideline-directed medications.

### Data sources

Patient-level data were obtained from the national SwedeHF registry [12]. Dispensation data including drug (Anatomic Therapeutic Chemical [ATC] code) and date of dispensation were obtained from the Swedish Prescribed Drug Register [13]. Mortality data for Swedish residents irrespective of citizenship or country of death were obtained from the Cause of Death Register [14], and used to determine the end of follow-up in patients who died during the study period.

### Study outcomes

Outcomes included all-cause and CV-related mortality. All hospital admissions were defined considering both main and sub-diagnoses from the inpatient visits.

### Main analysis: propensity score matching including clinical variables

Patients included in the main analyses were those recorded with an index registration in the SwedeHF registry between 1 April 2016 and 31 December 2020, were alive on 1 April 2016, had an LVEF < 50%, and had initiated ARNI ≤ 3 months before or after their index registration in SwedeHF or had at least one dispensation of ACEi/ARB (ATC code C09AA and C09CA, respectively) ≤ 3 months after their index registration. Propensity score matching was performed using exact matching on the propensity score estimate rounded to 0.005, with the 1:1 ARNI:ACEi/ARB ratio.

### Sensitivity analysis: propensity score matching including clinical variables

Two additional matching procedures, 1:2 and 1:3 ratios, were performed and comprised the sensitivity analysis for the propensity score approach. Methodological details for the sensitivity analyses and statistics used can be found in the Supporting Information.

## Results

### Patient population

**Fig. 1:**
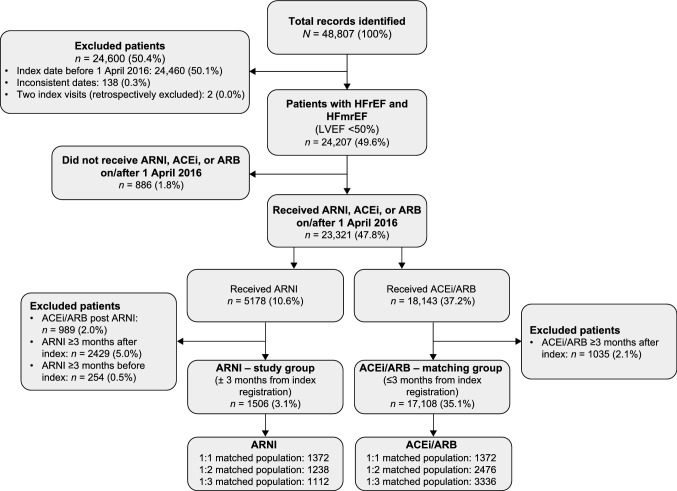
Flow diagram detailing the selection of patients used in propensity score matching. *ACEi* angiotensin-converting enzyme inhibitor, *ARB* angiotensin receptor blockers, *ARNI* angiotensin receptor–neprilysin inhibitor, *HFmrEF* heart failure with mildly reduced ejection fraction, *HFrEF* heart failure with reduced ejection fraction

In total, 48,807 patients identified through SwedeHF were alive on 1 April 2016 and had a LVEF < 50%. The flow diagram (Fig. 1) shows the details of inclusion and exclusion in this study, leaving 1506 patients in the ARNI study group and 17,108 in the ACEi/ARB matching group.

### Baseline characteristics

Baseline characteristics both pre- and post-matching for the ARNI and ACEi/ARB groups included in the main analysis cohort are summarized in Supplementary Table S1*.* Overall study cohort was well treated with recommended pharmacologic therapy for HFrEF including beta blocker (99%), MRA (70%), diuretics (73%), implantable cardioverter defibrillator (ICD) (24%), and CRT (14%) prior to initiation of ARNI. Approximately three-quarters of patients (76%) received prior treatment with ACEi/ARB. After 1:1 propensity score matching, 2744 patients were included in the primary analyses: 1372 patients in each treatment arm. Baseline characteristics including comorbidities were well balanced between groups and are summarized in Supplementary Table S1. Post-matching baseline characteristics were also well balanced after 1:2 and 1:3 matching, as summarized in the Supporting Information, Table S2.

### All-cause mortality with ARNI versus ACEi/ARB: propensity score matching including clinical variables

**Fig. 2:**
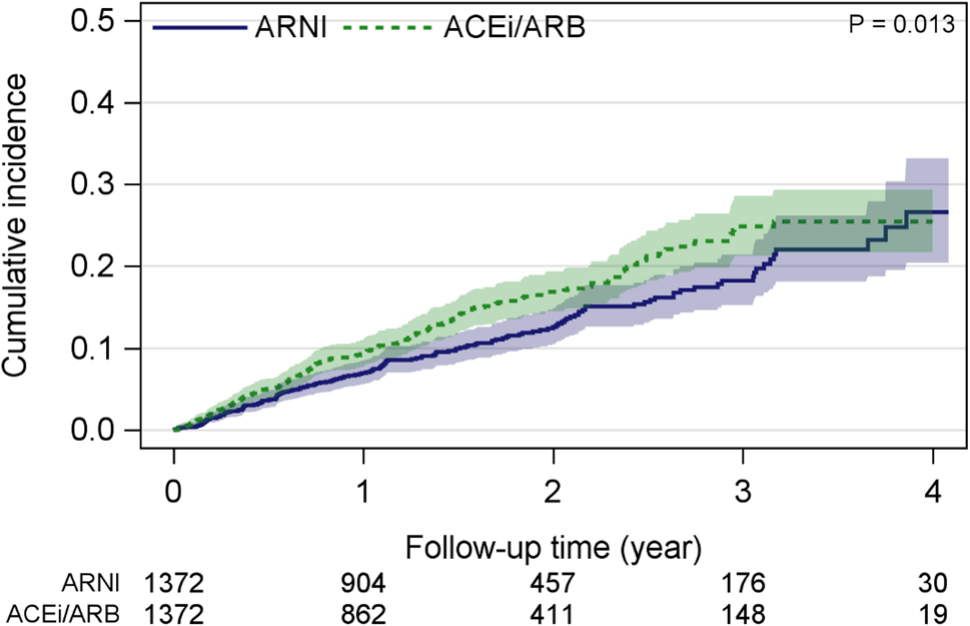
Cumulative incidence estimates with 95% confidence intervals for all-cause mortality for ARNI versus ACEi/ARB groups matched 1:1 ratio using propensity score matching including clinical variables. *ACEi* angiotensin-converting enzyme inhibitor, *ARB* angiotensin receptor blockers, *ARNI* angiotensin receptor–neprilysin inhibitor

ARNI was associated with a statistically significant 23% reduction in all-cause mortality compared with ACEi/ARB using 1:1 matching (HR 0.77 [95% CI 0.63–0.95], *p* = 0.013; Table 1). Estimated all-cause mortality for ARNI versus ACEi/ARB was 7.1 events (95% CI 6.1–8.3) versus 9.3 events per 100 person-years (95% CI 8.1–10.7; Table 1; Fig. 2). Outcomes were consistent in further sensitivity analysis with 1:2 and 1:3 matching (Supporting Information, Table S3, Fig. S1). Similar outcomes were obtained following stratification of the 1:1 matching groups as follows: ≤ median NT-proBNP (HR 0.78 [95% CI 0.55–1.10]; *p* = 0.15); > median NT-proBNP (HR 0.77 [95% CI 0.59–1.00]; *p* = 0.049); HFmrEF (HR 0.75 [95% CI 0.37–1.53]; *p* = 0.43]), and HFrEF (HR 0.77 [95% CI 0.62–0.96]; *p* = 0.020]).

### Cardiovascular-related mortality with ARNI versus ACEi/ARB: propensity score matching including clinical variables

**Fig. 3:**
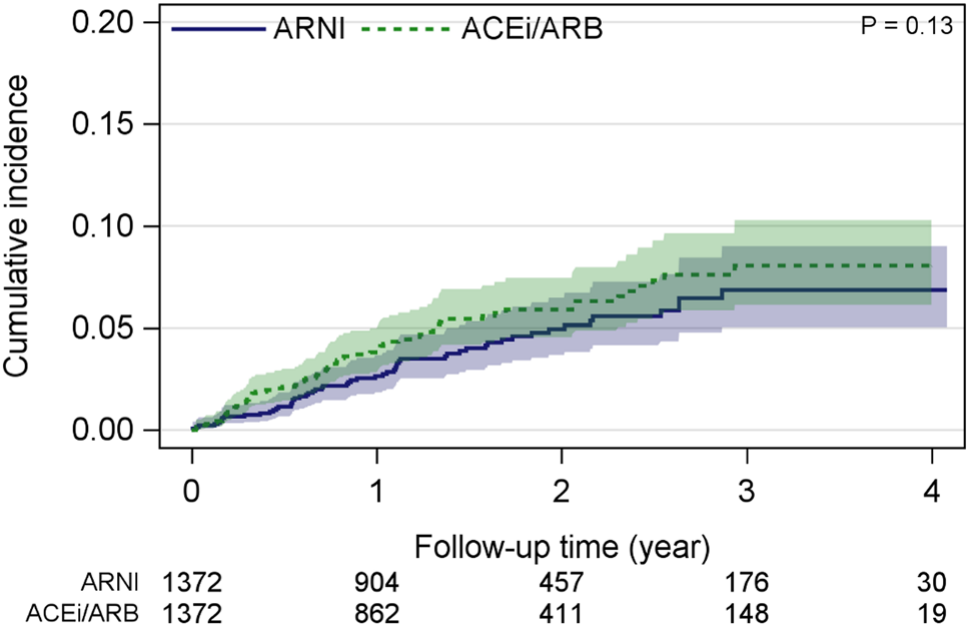
Cumulative incidence estimates with 95% confidence intervals for cardiovascular-related mortality (handling other death as competing risk) for ARNI versus ACEi/ARB groups matched 1:1 ratio using propensity score matching including clinical variables. *ACEi* angiotensin-converting enzyme inhibitor, *ARB* angiotensin receptor blockers, *ARNI* angiotensin receptor–neprilysin inhibitor

ARNI was associated with a non-significant 23% risk reduction in CV-related mortality using 1:1 matching, compared with ACEi/ARB (HR 0.77 [95% CI 0.54–1.09], *p* = 0.13; Table 1). Estimated CV-related mortality per 100 person-years for ARNI versus ACEi/ARB was: 2.5 (95% CI 1.9–3.3) versus 3.3 (95% CI 2.6–4.2) (Table 1; Fig. 3*)*. Similar results were obtained for the 1:2 and 1:3 sensitivity analysis matchings (Supporting Information, Table S3, Fig. S2).

### Hospitalizations with ARNI versus ACEi/ARB: propensity score matching including clinical variables

There was no difference in the risk of all-cause and CV-related hospitalization between groups. The relative risks of all-cause and CV-related hospitalization with ARNI versus ACEi/ARB were, respectively: 1.02 (95% CI 0.91–1.13, *p* = 0.76) and 1.01 (95% CI 0.91–1.15, *p* = 0.84) using 1:1 matching (Table 1). Similar results were obtained in the sensitivity analyses (Supporting Information, Table S3).

**Table 1:** Main analysis: Real-world all-cause and cardiovascular-related mortality, and all-cause and cardiovascular-related hospitalization using ARNI:ACEi/ARB in propensity score 1:1 ratio matching including clinical variables

Endpoint	ARNI			ACEi/ARB			ARNI vs ACEi/ARB	
	Duration of follow-up, years, median (IQR)	Events, *n*/*N* (%)	Event rate per 100 person-years (95% CI)	Duration of follow-up, years, median (IQR)	Events, *n*/*N* (%)	Event rate per 100 person-years (95% CI)	HR (95% CI)	*p* value
All-cause mortality								
1:1 PS matched	1.5 (0.8–2.3)	160/1372 (11.7)	7.1 (6.1–8.3)	1.4 (0.8–2.3)	200/1372 (14.6)	9.3 (8.1–10.7)	0.77 (0.63–0.95)	0.013
Cardiovascular-related mortality								
1:1 PS matched	1.5 (0.8–2.3)	57/1372 (4.2)	2.5 (1.9–3.3)	1.4 (0.8–2.3)	72/1372 (5.2)	3.3 (2.6–4.2)	0.77 (0.54–1.09)	0.13
All-cause hospitalization								
1:1 PS matched	0.7 (0.3–1.5)	671/1372 (48.9)	47.9 (44.3–51.6)	0.7 (0.2–1.6)	649/1372 (47.3)	46.5 (43.0–50.2)	1.02 (0.91–1.13)	0.76
Cardiovascular-related hospitalization								
1:1 PS matched	0.8 (0.3–1.6)	643/1372 (46.9)	44.5 (41.2–48.1)	0.8 (0.3–1.6)	624/1372 (45.5)	43.8 (40.4–47.3)	1.01 (0.91–1.15)	0.84

*ACEi* angiotensin-converting enzyme inhibitor, *ARB* angiotensin receptor blockers, *ARNI* angiotensin receptor–neprilysin inhibitor, *CI* confidence interval, *HR* hazard ratio, *IQR* interquartile range, *PS* propensity score

### Sensitivity analysis: exact matching without clinical variables

Of the 48,807 patients who were alive and identified through SwedeHF, 48,667 (99.7%) were eligible for inclusion in the sensitivity analysis (Supporting Information, Fig. S3). A total of 4125 (8.5%) patients were excluded owing to no dispensation of ARNI/ACEi/ARB on or after 1 April 2016. Overall, 7275 patients (14.9%) received ARNI and 37,267 (76.4%) received ACEi/ARB only. A total of 1518 (3.1%) patients who received ARNI were excluded because they received a dispensation for ACEi/ARB following ARNI, and the remaining 5757 patients (11.8%) were available for exact matching 1:2 procedure using sex, age, HF duration, and index year as matching variables. This sensitivity analysis compared 4791 (9.8%) patients in the ARNI group and 9582 (19.6%) in the ACEi/ARB group. In both groups, 79.6% were male, mean age was 68.8 years, and 46.5% had a duration of HF ≥ 6 months at the index visit. Baseline characteristics are included in Supporting Information, Table S4. ARNI was associated with a statistically significant 10% reduction in all-cause mortality compared with ACEi/ARB (HR 0.90 [95% CI 0.82–0.99], *p* = 0.026), but no difference in CV-related mortality (HR 1.04 [95% CI 0.89–1.22], *p* = 0.63; Supporting Information Table S3 and Fig. S4).

## Discussion

We have demonstrated that, in line with the pivotal PARADIGM-HF trial, the use of ARNI in a real-world nationwide Swedish HF population was associated with a statistically significant risk reduction in all-cause mortality in patients with HFrEF or HFmrEF (LVEF < 50%) compared with ACEi/ARB. To our knowledge, this is the first European study to publish such findings, which are leveraged by the unique infrastructure of the Swedish national registries that facilitate the linking of granular patient-level clinical data to nationwide health databases with pharmacy dispensation, resource utilization, and mortality data [14]. Propensity score matching methodology ensured that patient baseline characteristics were generally well balanced between treatment arms, thus reducing confounding factors.

Real-world patient demographics in this study differ from those enrolled in the PARADIGM-HF trial. In line with Fu et al. [3], patients in our study who received ARNI as part of real-world Swedish clinical practice were older, had more severe disease, and a higher incidence of atrial fibrillation and stroke than those enrolled in the multinational PARADIGM-HF trial [1], which included nine sites in Sweden [15]. Despite the differences in patient demographics, in the real world, patients receiving ARNI experienced a 23% significant reduction in risk of all-cause mortality compared with ACEi/ARB, which is consistent with the statistically significant 16% reduction in all-cause mortality demonstrated in the PARADIGM-HF trial [1]. We also revealed a 23% reduction in CV-related mortality with ARNI compared with ACEi/ARB in line with the 20% reduction in risk of CV-related death reported in the PARADIGM-HF trial. However, our findings on CV-related mortality failed to reach statistical significance, possibly owing to the lower event rate for CV-related mortality (2.5 [95% CI 1.9–3.3] per 100 person-years) than all-cause mortality (7.1 [95% CI 6.1–8.3] per 100 person-years). The most probable explanation as to the lower event rate for CV-related mortality in our study is the high usage of optimal background therapy including beta blocker (99%), MRA (70%), diuretics (73%), prior treatment with ACEi/ARB (76%), ICD (24%), and CRT (14%) prior to initiation of ARNI. Moreover, variations in International Classification of Diseases 10th Revision (ICD-10) coding for cause of death in the real-world setting may have introduced further uncertainty into the analysis. While clinical trials such as PARADIGM-HF follow a predefined protocol with strict adjudication by an endpoint committee, in the real-world setting, the coding is at the discretion of the treating physician. This may also explain, to some extent, the lack of difference in hospitalization risk between treatment groups in our study, which differs from the outcomes reported in PARADIGM-HF.

Interestingly, Tan et al*.,* who similarly reported a reduction in risk of all-cause mortality (20% reduction; *p* = 0.027) and hospitalization (14% reduction; *p* < 0.001) with ARNI compared with ACEi/ARB in a US-based comparative effectiveness propensity scoring analysis, also cited discrepancies in ICD-10 coding as a possible explanation for the lack of difference in HF-related hospitalization reported in their study [16]. However, in our study, we observed no differences in either risk of all-cause or CV-related hospitalizations between the two treatment groups with similar results seen in the sensitivity analysis. The lack of difference in hospitalization risk between the two treatment groups in our study is likely to reflect a real difference between clinical trials, where protocols detailing the adjudication of events are available, and routine clinical practice. Whether this is true, or only in Sweden, needs to be further studied.

Although the real-world effectiveness of ARNI versus ACEi has previously been demonstrated, our study is not only the first to report the comparative effectiveness on all-cause mortality in a European population, but unlike other registry studies, it utilized the unique infrastructure of the Swedish datasets, which allowed us to obtain granular patient-level clinical data linked to other national health registries. Our study also included a small proportion of patients (9.5%) with LVEF 40–49% (i.e., HFmrEF), and is, to our knowledge, the first real-world evidence study to do so. *Post hoc* analyses stratified by LVEF confirmed a similar reduction in relative risk of all-cause mortality in patients with LVEF < 40% (i.e., HFrEF; 23% reduction [*p* = 0.020]) or LVEF 40–49% (i.e., HFmrEF; 25% numerical reduction; [*p* = 0.43]), further supporting the concept that HF should be viewed as a continuous variable [17]. The subpopulation with HFmrEF was small; however, these outcomes are aligned with a pooled analysis of data from PARADIGM-HF and PARAGON-HF [18]. A previous lack of prospective evidence, and of guideline-directed medical therapy has meant that ARNI is not currently widely used for the treatment of HFmrEF in clinical practice, further highlighting the relevance of our study.

Eligibility for ARNI in Swedish clinical practice requires patients to have exhausted all alternative treatment options, which may account for both the higher prior MRA and ACEi/ARB use, and increased longevity of disease observed in the ARNI group compared with the ACEi/ARB group [3]. Clinicians may also be hesitant to prescribe owing to questions surrounding the clinical applicability of the results of the PARADIGM-HF trial to the Swedish HF population. The long run-in period (6–8 weeks) prior to randomization when patients were required to receive single-blind enalapril followed by single-blind ARNI saw 12% of patients discontinue treatment owing to adverse events, and thus could be argued as a period of patient selection.

The strength of this study hinges on the use of large national registries, the structure of which enabled identification of data on treatment dispensation, hospitalization, and mortality to be obtained for patients with HFrEF or HFmrEF identified through the SwedeHF registry with a median follow-up of approximately 1.5 years. The availability of patient-level data for a large cohort allowed the use of carefully considered propensity score matching methodology to match key clinical variables, which to our knowledge cannot be captured to the same extent in other databases. The primary analysis was confirmed by the sensitivity analysis, and was consistent with the published literature. Indeed, the US-based propensity scoring analysis conducted by Tan et al*.* and a meta-analysis published by Proudfoot et al*.* that included the study by Tan et al. also showed a statistically significant reduction in all-cause mortality with ARNI versus ACEi/ARB; however, no comparative effectiveness study on outcomes in a European population was available for inclusion in this publication [7, 16].

As is the nature of retrospective observational studies, there are limitations. Our desire to use the granularity of the clinical data from SwedeHF meant that not all patients initiated ARNI at the time they were reported to SwedeHF. To address this, we limited patients to those who received ARNI 3 months on either side of the index date; however, variability may still have been introduced within this window, additionally it resulted in a smaller sample size with the exclusion of approximately 70% of eligible patients from the main analysis. The impact of the reduction in sample size was tested in a sensitivity analysis that used exact matching without clinical variables, and included 66% of all patients who received ARNI in Sweden over the study period. In addition, patients were not continuously followed up throughout the study period, which presents a further limitation to the methodology potentially contributing to variability within the study. As with many observational studies, key lifestyle evidence (e.g., smoking) was not captured in the databases used, and socioeconomic data were not included in our analysis, meaning that there was no adjustment for these potentially confounding factors.

In conclusion, this nationwide real-world study indicates that a population of patients with HFrEF and HFmrEF who received ARNI as part of guideline-led Swedish clinical practice were associated with a statistically significant relative risk reduction in all-cause mortality compared with ACEi/ARB, in line with the mortality outcomes of the randomized PARADIGM-HF trial [8]. However, in contrast with previous randomized controlled trials, no difference was observed in the rate of hospitalization in real-world treatment with ARNI compared with ACEi/ARB in Sweden. In line with the newly issued 2021 ESC HF guideline update, in which ARNI is recommended for the management of HFrEF and HFmrEF, our findings are timely to enhance the understanding of the effectiveness of ARNI in routine clinical practice in Sweden.

### Electronic supplementary material

Below is the link to the electronic supplementary material.

Supplementary file1 (DOCX 673 kb)

## Appendix

See supporting material.
