# Supplementary material for: Real-world comparative effectiveness of ARNI versus ACEi/ARB in HF with reduced or mildly reduced ejection fraction
Source: Clin Res Cardiol. 2022 Nov 29;112(1):167–74. doi: 10.1007/s00392-022-02124-w (PMC9849288; doi:10.1007/s00392-022-02124-w)

# Supplementary files

## Sensitivity analysis: exact matching without clinical variables

Patients included in the sensitivity analysis were those recorded in the SwedeHF registry who were alive on 1 April 2016, had an LVEF < 50%, and had received at least one dispensation of ARNI or had at least one dispensation of ACEi/ARB between 1 April 2016 and 31 December 2020. Combined treatment with ARNI and ARB was not permitted, nor was sequential use of ARNI followed by ACEi/ARB.

Sensitivity analyses were performed to test the consistency of the data and robustness of the primary analysis. Exact 1:2 matching without clinical variables was performed for each calendar year between 2016 and 2020. This was based on the date of treatment initiation in patients receiving ARNI, and a hypothetical randomly selected month in the same year for those who received ACEi/ARB. Patients selected for the ACEi/ARB group were confirmed alive and receiving medication on day 1 of the hypothetical randomly selected month before the exact matching was performed using the following variables collected at the index registration: sex, age, HF duration (< 6/≥ 6 months), and index year. Patients receiving ARNI were followed from the date of treatment, and those receiving ACEi/ARB from the randomly selected starting month. Both groups were followed up until the studied outcome, death, or 31 December 2020.

## Statistics

Continuous variables are presented as mean and standard deviation, or median, minimum, and maximum, and categorical variables as number and percentage. The incidence of all-cause and CV-related mortality was described using crude event rates per 100 person-years with exact Poisson 95% confidence intervals (CIs), hazard ratios (HRs; Cox regression) with 95% CIs, and log-rank test. Analyses stratified by NT-proBNP (≤ median, > median) and by ejection fraction (40–49% [HFmrEF], < 40% [HFrEF]) were performed *post hoc* on 1:1 matching groups. The proportional hazards assumption was examined by visual review of log(–log(survival)) versus log(follow-up time) curves. Cumulative incidence curves were produced for all study outcomes; for CV mortality and hospitalizations, other death and all-cause death, respectively, were considered to be competing risk.[22] For tests between the ARNI and ACEi/ARB groups with respect to baseline data, Fisher’s exact test was used for dichotomous variables, Mantel–Haenszel chi-square trend test for ordered categorical variables, and Mann–Whitney U-test for continuous variables; tests were two-tailed and evaluated on 0.05 significance level. All analyses were performed using SAS software version 9.4 (SAS Institute Inc., Cary, NC, USA).

## Supplementary table S1 Clinical and medical characteristics for patients who received ARNI ±3 months or ACEi/ARB ≤ 3 months from index registration in SwedeHF pre- and post-1:1 propensity score matching

| **Variable** | **Pre-matching** | | | **Post-matching (1:1)** | | |
| --- | --- | --- | --- | --- | --- | --- |
|  | **ARNI (*n*=1506)** | **ACEi/ARB (*n*=17,108)** | ***p* value^a^** | **ARNI (*n*=1372)** | **ACEi/ARB (*n*=1372)** | ***p* value^a^** |
| Sex, male, n (%)^b^ | 1186 (78.8) | 11,488 (67.1) | < 0.0001 | 1062 (77.4) | 1089 (79.4) | 0.23 |
| Age at index, years, mean ± SD^b^ | 67.8 ± 12.2 | 72.7 ± 12.0 | < 0.0001 | 68.2 ± 12.2 | 68.6 ± 12.9 | 0.25 |
| HF duration ≥ 6 months, n (%)^b^ | 894 (60.2) | 6273 (38.1) | < 0.0001 | 781 (57.8) | 768 (56.6) | 0.58 |
| Inpatient care, n (%)^b^ | 152 (10.1) | 4355 (25.5) | < 0.0001 | 148 (10.8) | 144 (10.5) | 0.85 |
| Body mass index, kg/m^2^, n (%)^b^ | *n*=1263 | *n*=13512 |  | *n*=1143 | *n*=1145 |  |
| < 18.5 | 11 (0.9) | 281 (2.1) | < 0.0001 | 11 (1.0) | 8 (0.7) | 0.81 |
| 18.5–25 | 361 (28.6) | 4656 (34.5) |  | 335 (29.4) | 337 (29.4) |  |
| > 25–30 | 479 (37.9) | 4999 (37.0) |  | 425 (37.2) | 444 (38.8) |  |
| > 30–35 | 267 (21.1) | 2369 (17.5) |  | 243 (21.3) | 226 (19.7) |  |
| > 35 | 145 (11.5) | 1207 (8.9) |  | 129 (11.3) | 130 (11.4) |  |
| Systolic blood pressure, mmHg, mean ± SD^c^ | *n*=1340 | *n*=12,506 |  | *n*=1211 | *n*=1201 |  |
|  | 120.4 ± 18.5 | 128.0 ± 20.7 | < 0.0001 | 121.0 ± 18.5 | 124.8 ± 20.8 | < 0.0001 |
| Diastolic blood pressure, mmHg, mean ± SD^c^ | n=1336 | n=12470 |  | *n* = 1207 | *n* = 1193 |  |
|  | 73.0 ± 11.5 | 74.9 ± 12.2 | <0.0001 | 73.3 ± 11.5 | 74.9 ± 12.7 | 0.0094 |
| NYHA functional class, *n* (%)^b^ | *n* = 1353 | *n* = 12678 |  | *n* = 1227 | *n* = 1230 |  |
| I | 95 (7.0) | 1653 (13.0) | <0.0001 | 95 (7.7) | 104 (8.5) | 0.52 |
| II | 626 (46.3) | 6595 (52.0) |  | 576 (46.9) | 577 (46.9) |  |
| III | 609 (45.0) | 4258 (33.6) |  | 535 (43.6) | 532 (43.3) |  |
| IV | 23 (1.7) | 172 (1.4) |  | 21 (1.7) | 17 (1.4) |  |
| LVEF, %, *n* (%)^b^ |  |  | <0.0001 |  |  | 0.72 |
| 40–<50 | 130 (8.6) | 6294 (36.8) |  | 130 (9.5) | 115 (8.4) |  |
| 30–<40 | 634 (42.1) | 6339 (37.1) |  | 590 (43.0) | 632 (46.1) |  |
| <30 | 742 (49.3) | 4475 (26.2) |  | 652 (47.5) | 625 (45.6) |  |
| NT-proBNP, pg/ml, median (min., max.)^b^ | *n* = 1252 | *n* = 13083 | 0.0001 | *n* = 1134 | *n* = 1144 | 0.78 |
|  | 1813 (42, 47 052) | 2150 (6, 122 000) |  | 1873 (42, 47 052) | 1867 (19, 122 000) |  |
| Comorbidities, *n* (%)^b^ |  |  |  |  |  |  |
| No ischaemic aetiology | 663 (55.2) | 7637 (58.9) | 0.014 | 617 (56.6) | 613 (55.4) | 0.61 |
| Hypertension | 1060 (70.4) | 11859 (69.3) | 0.41 | 973 (70.9) | 960 (70.0) | 0.62 |
| Atrial fibrillation | 769 (51.1) | 9001 (52.6) | 0.26 | 709 (51.7) | 700 (51.0) | 0.76 |
| COPD | 223 (14.8) | 3029 (17.7) | 0.0044 | 214 (15.6) | 214 (15.6) | 1.00 |
| Diabetes mellitus | 495 (32.9) | 4261 (24.9) | <0.0001 | 450 (32.8) | 436 (31.8) | 0.60 |
| Blood diseases | 279 (18.5) | 3449 (20.2) | 0.14 | 251 (18.3) | 246 (17.9) | 0.84 |
| Stroke/TIA | 225 (14.9) | 2266 (13.2) | 0.073 | 200 (14.6) | 191 (13.9) | 0.66 |
| Psychiatric diagnoses^d^ | 171 (11.4) | 2396 (14.0) | 0.0040 | 162 (11.8) | 164 (12.0) | 0.95 |
| Musculoskeletal diseases^d^ | 205 (13.5) | 2951 (17.2) | 0.0003 | 191 (13.9) | 205 (14.9) | 0.48 |
| Malignant cancer^d^ | 136 (9.0) | 1950 (11.4) | 0.0049 | 129 (9.4) | 126 (9.2) | 0.90 |
| Treatments, *n* (%) |  |  |  |  |  |  |
| Beta blockers | 1426 (99.2) | 15,512 (97.4) | < 0.0001 | 1301 (99.2) | 1285 (99.2) | 1.00 |
| Diuretics | 1072 (72.8) | 10,310 (68.0) | 0.0002 | 978 (73.0) | 992 (73.9) | 0.66 |
| Mineralocorticoid receptor antagonists^b^ | 1055 (70.1) | 6521 (38.2) | <0.0001 | 932 (68.0) | 935 (68.2) | 0.96 |
| Implantable cardiovascular device^b^ | 353 (23.5) | 816 (4.8) | < 0.0001 | 246 (18.0) | 227 (16.6) | 0.36 |
| Cardiac resynchronization therapy^b^ | 208 (13.8) | 575 (3.4) | < 0.0001 | 150 (10.9) | 160 (11.7) | 0.59 |
| ACEi/ARB prior to 3 months before index date, *n* (%) | 1150 (76.4) | 9415 (55.0) | < 0.0001 | 1028 (74.9) | 918 (66.9) | < 0.0001 |
| Time from index date to initiation of ARNI, years, median (min., max.) | –0.04  (–0.25, 0.24) | N/A |  | –0.04  (–0.25, 0.24) | N/A |  |

ACEi, angiotensin-converting enzyme inhibitor; ARB, angiotensin receptor blockers; ARNI, angiotensin receptor–neprilysin inhibitor; COPD, chronic obstructive pulmonary disease; HF, heart failure; LVEF, left ventricular ejection fraction; max., maximum; min., minimum; N/A, not applicable; NT-proBNP, N-terminal pro B-type natriuretic peptide; NYHA, New York Heart Association; SD, standard deviation; SwedeHF, Swedish Heart Failure Registry; TIA, transient ischaemic attack.

^a^For comparison between groups, Fisher’s Exact test (lowest one-sided *p* value multiplied by 2) was used for dichotomous variables, the Mantel–Haenszel Chi-square test was used for ordered categorical variables, and the Mann–Whitney U-test was used for continuous variables.

^b^Variable used in matching.

^c^Outpatients only.

^d^In the 3 years before admission.

## Supplementary Table S2 Clinical and medical characteristics for patients who received ARNI ±3 months or ACEi/ARB ≤3 months from index registration in SwedeHF following 1:2 and 1:3 propensity score matching for sensitivity analysis.

| **Variable** | **1:2 matching** | | | **1:3 matching** | | |
| --- | --- | --- | --- | --- | --- | --- |
|  | **ARNI (*n* = 1238)** | **ACEi/ARB (*n* = 2476)** | ***P* value^a^** | **ARNI (*n* = 1112)** | **ACEi/ARB (*n* = 3336)** | ***P* value^a^** |
| Sex, male, n (%) | 943 (76.2) | 1883 (76.1) | 0.97 | 842 (75.7) | 2553 (76.5) | 0.61 |
| Age at index, years, mean ± SD | 68.8 ± 12.0 | 68.8 ± 13.1 | 0.59 | 69.0 ± 12.1 | 69.4 ± 12.8 | 0.24 |
| Inpatient care, n (%) | 144 (11.6) | 293 (11.8) | 0.90 | 138 (12.4) | 395 (11.8) | 0.65 |
| HF duration ≥6 months, n (%) | 670 (55.1) | 1316 (54.1) | 0.62 | 577 (52.8) | 1695 (51.7) | 0.54 |
| Body mass index, kg/m^2^, n, (%) | *n* = 1029 | *n* = 2057 |  | *n* = 924 | *n* = 2759 |  |
| <18.5 | 11 (1.1) | 21 (1.0) | 0.42 | 11 (1.2) | 31 (1.1) | 0.85 |
| 18.5–25 | 308 (29.9) | 638 (31.0) |  | 281 (30.4) | 830 (30.1) |  |
| >25–30 | 388 (37.7) | 782 (38.0) |  | 350 (37.9) | 1052 (38.1) |  |
| >30–35 | 216 (21.0) | 418 (20.3) |  | 188 (20.3) | 563 (20.4) |  |
| >35 | 106 (10.3) | 198 (9.6) |  | 94 (10.2) | 283 (10.3) |  |
| Systolic blood pressure, mmHg, mean ± SD^b^ | *n* = 1082 | *n* = 2139 |  | *n* = 963 | *n* = 2893 |  |
|  | 121.3 ± 18.7 | 125.4 ± 20.8 | <0.0001 | 121.4 ± 18.6 | 125.0 ± 20.6 | <0.0001 |
| Diastolic blood pressure, mmHg, mean ± SD^b^ | *n* = 1079 | *n* = 2128 |  | *n* = 961 | *n* = 2879 |  |
|  | 73.3 ± 11.5 | 75.1 ± 12.2 | <0.0001 | 73.2 ± 11.5 | 74.9 ± 12.6 | 0.0004 |
| NYHA functional class, n (%) | *n* = 1095 | *n* = 2186 |  | *n* = 980 | *n* = 2933 |  |
| I | 91 (8.3) | 171 (7.8) | 0.99 | 85 (8.7) | 219 (7.5) | 0.95 |
| II | 512 (46.8) | 1045 (47.8) |  | 455 (46.4) | 1428 (48.7) |  |
| III | 475 (43.4) | 935 (42.8) |  | 421 (43.0) | 1238 (42.2) |  |
| IV | 17 (1.6) | 35 (1.6) |  | 19 (1.9) | 48 (1.6) |  |
| LVEF, n (%) |  |  |  |  |  |  |
| 40–<50 | 129 (10.4) | 244 (9.9) | 0.97 | 125 (11.2) | 339 (10.2) | 0.56 |
| 30–<40 | 541 (43.7) | 1112 (44.9) |  | 488 (43.9) | 1492 (44.7) |  |
| <30 | 568 (45.9) | 1120 (45.2) |  | 499 (44.9) | 1505 (45.1) |  |
| NT-proBNP, pg/ml, median (95 CI) | *n* = 1023 | *n* = 2066 |  | *n* = 920 | *n* = 2749 |  |
|  | 1940 (42–47 052) | 2049 (14–122 000) |  | 1973 (42–47 052) | 2066 (19– 122 000) |  |
| Comorbidities, n (%) |  |  |  |  |  |  |
| No ischaemic aetiology | 561 (57.0) | 1171 (59.7) | 0.17 | 511 (57.9) | 1489 (57.1) | 0.68 |
| Hypertension | 877 (70.8) | 1742 (70.4) | 0.79 | 777 (69.9) | 2345 (70.3) | 0.82 |
| Atrial fibrillation | 648 (52.3) | 1279 (51.7) | 0.72 | 581 (52.2) | 1744 (52.3) | 1.00 |
| COPD | 203 (16.4) | 364 (14.7) | 0.19 | 176 (15.8) | 495 (14.8) | 0.45 |
| Diabetes mellitus | 395 (31.9) | 767 (31.0) | 0.59 | 342 (30.8) | 1000 (30.0) | 0.65 |
| Blood diseases | 230 (18.6) | 472 (19.1) | 0.76 | 210 (18.9) | 627 (18.8) | 0.98 |
| Stroke/TIA | 182 (14.7) | 358 (14.5) | 0.88 | 156 (14.0) | 456 (13.7) | 0.80 |
| Psychiatric diagnoses^c^ | 142 (11.5) | 297 (12.0) | 0.68 | 140 (12.6) | 387 (11.6) | 0.41 |
| Musculoskeletal diseases^c^ | 179 (14.5) | 360 (14.5) | 0.99 | 164 (14.7) | 470 (14.1) | 0.62 |
| Malignant cancer^c^ | 121 (9.8) | 240 (9.7) | 0.98 | 114 (10.3) | 336 (10.1) | 0.90 |
| Treatments |  |  |  |  |  |  |
| Beta blockers | 1175 (99.1) | 2333 (99.3) | 0.64 | 1051 (99.1) | 3139 (99.2) | 0.68 |
| Diuretics | 881 (73.1) | 1770 (73.2) | 1.00 | 788 (73.0) | 2371 (72.9) | 0.95 |
| Mineralocorticoid receptor antagonists | 812 (65.7) | 1645 (66.6) | 0.61 | 706 (63.6) | 2121 (63.7) | 1.00 |
| Implantable cardiovascular device | 184 (14.9) | 351 (14.2) | 0.60 | 144 (13.0) | 398 (12.0) | 0.40 |
| Cardiac resynchronization therapy | 115 (9.3) | 227 (9.2) | 0.94 | 98 (8.8) | 271 (8.1) | 0.51 |

ACEi, angiotensin-converting enzyme inhibitor; ARB, angiotensin receptor blockers; ARNI, angiotensin receptor–neprilysin inhibitor; bpm, beats per minute; CI, confidence interval; COPD, chronic obstructive pulmonary disease; HF, heart failure; LVEF, left ventricular ejection fraction; NT-proBNP, N-terminal pro B-type natriuretic peptide; NYHA, New York Heart Association; SD, standard deviation; TIA, transient ischaemic attack.

^a^For comparison between groups, Fisher’s Exact test (lowest 1-sided *P* value multiplied by 2) was used for dichotomous variables, the Mantel–Haenszel Chi-square test was used for ordered categorical variables, and the Mann–Whitney U-test was used for continuous variables.

^b^Outpatients only.

^c^In the 3 years before admission.

## Supplementary Table S3 Sensitivity analyses, real-world all-cause and cardiovascular-related mortality, and all-cause and cardiovascular-related hospitalization using ARNI:ACEi/ARB ratios 1:2, and 1:3 in propensity score matching including clinical variables and using 1:2 exact matching without clinical variables

| **Endpoint** | **ARNI** | | | **ACEi/ARB** | | | **ARNI vs ACEi/ARB** | | |
| --- | --- | --- | --- | --- | --- | --- | --- | --- | --- |
|  | **Duration of follow-up, years, median (IQR)** | **Events, *n*/*N* (%)** | **Event rate per 100-person-years (95% CI)** | **Duration of follow-up, years, median (IQR)** | **Events, *n*/*N* (%)** | **Event rate per 100-person-years (95% CI)** | **HR (95% CI)** | ***P-*value** | |
| All-cause mortality | | | | | | | | |  |
| 1:2 PS matched | 1.5 (0.8–2.3) | 151/1238 (12.2) | 7.4 (6.3–8.7) | 1.4 (0.8–2.3) | 367/2476 (14.8) | 9.2 (8.3–10.2) | 0.80 (0.67–0.97) | 0.024 | |
| 1:3 PS matched | 1.5 (0.8–2.4) | 136/1112 (12.2) | 7.4 (6.2–8.7) | 1.5 (0.8–2.3) | 500/3336 (15.0) | 9.2 (8.4–10.0) | 0.80 (0.67–0.97) | 0.024 | |
| Exact matching (1:2) | 1.5 (0.8–2.3) | 619/4791 (12.9) | 8.0 (7.4–8.6) | 1.5 (0.7, 2.4) | 1389/9582 (14.5) | 8.9 (8.4–9.4) | 0.90 (0.82–0.99) | 0.026 | |
| Cardiovascular-related mortality | | | | | | | | |  |
| 1:2 PS matched | 1.5 (0.8–2.3) | 55/1238 (4.4) | 2.7 (2.0–3.5) | 1.4 (0.8–2.3) | 133/2476 (5.4) | 3.3 (2.8–4.0) | 0.81 (0.59–1.11) | 0.19 | |
| 1:3 PS matched | 1.5 (0.8–2.4) | 52/1112 (4.7) | 2.8 (2.1–3.7) | 1.5 (0.8–2.3) | 185/3336 (5.5) | 3.4 (2.9–3.9) | 0.83 (0.61–1.13) | 0.25 | |
| Exact matching (1:2) | 1.5 (0.8–2.3) | 234/4791 (4.9) | 3.0 (2.6–3.4) | 1.5 (0.7–2.4) | 452/9582 (4.7%) | 2.9 (2.6–3.2) | 1.04 (0.89–1.22) | 0.63 | |
| All-cause hospitalization | | | | | | | | |  |
| 1:2 PS matched | 0.7 (0.3–1.5) | 622/1238 (50.2) | 49.6 (45.8–53.6) | 0.8 (0.3–1.6) | 1183/2476 (47.8) | 46.5 (43.9–49.2) | 1.05 (0.96–1.16) | 0.29 | |
| 1:3 PS matched | 0.8 (0.3–1.5) | 564/1112 (50.7) | 49.9 (45.8–54.2) | 0.8 (0.2–1.6) | 1626/3336 (48.7) | 47.6 (45.3–49.9) | 1.04 (0.94–1.14) | 0.47 | |
| Cardiovascular-related hospitalization | | | | | | | | |  |
| 1:2 PS matched | 0.8 (0.3–1.6) | 594/1238 (48.0) | 45.9 (42.2–49.7) | 0.8 (0.3–1.6) | 1126/2476 (45.5) | 42.9 (40.4–45.5) | 1.06 (0.96–1.17) | 0.27 | |
| 1:3 PS matched | 0.8 (0.3–1.5) | 542/1112 (48.7) | 46.6 (42.8–50.7) | 0.8 (0.3–1.6) | 1567/3336 (47.0) | 44.7 (42.5–47.0) | 1.03 (0.94–1.14) | 0.51 | |

ACEi, angiotensin-converting enzyme inhibitor; ARB, angiotensin receptor blockers; ARNI, angiotensin receptor–neprilysin inhibitor; CI, confidence interval; HR, hazard ratio; IQR, interquartile range; PS, propensity score.

## Supplementary Table S4 Patient baseline characteristics for the cohort used in exact 1:2 matching without clinical variables

| **Variable** | **ARNI (*n* = 4791)** | **ACEi/ARB (*n* = 9582)** | ***P*value** |
| --- | --- | --- | --- |
| Sex, male, n (%) | 3815 (79.6) | 7630 (79.6) | 1.00 |
| Age at index, years, mean ± SD | 68.8 ± 9.9 | 68.8 ± 9.9 | 0.94 |
| HF duration ≥6 months, n (%) | 2227 (46.5) | 4454 (46.5) | 1.00 |
| Index year, n (%) |  |  |  |
| 2004 | 7 (0.1) | 14 (0.1) | 1.00 |
| 2005 | 17 (0.4) | 34 (0.4) |  |
| 2006 | 45 (0.9) | 90 (0.9) |  |
| 2007 | 62 (1.3) | 124 (1.3) |  |
| 2008 | 96 (2.0) | 192 (2.0) |  |
| 2009 | 103 (2.1) | 206 (2.1) |  |
| 2010 | 98 (2.0) | 196 (2.0) |  |
| 2011 | 133 (2.8) | 266 (2.8) |  |
| 2012 | 146 (3.0) | 292 (3.0) |  |
| 2013 | 143 (3.0) | 286 (3.0) |  |
| 2014 | 194 (4.0) | 388 (4.0) |  |
| 2015 | 186 (3.9) | 372 (3.9) |  |
| 2016 | 374 (7.8) | 748 (7.8) |  |
| 2017 | 697 (14.5) | 1394 (14.5) |  |
| 2018 | 883 (18.4) | 1766 (18.4) |  |
| 2019 | 1006 (21.0) | 2012 (21.0) |  |
| 2020 | 601 (12.5) | 1202 (12.5) |  |

For categorical variables, n (%) is presented. For comparison between groups, Fisher’s Exact test (lowest 1-sided *P* value multiplied by 2) was used for dichotomous variables, the Mantel–Haenszel Chi-square test was used for ordered categorical variables, and the Mann–Whitney U-test was used for continuous variables.

ACEi, angiotensin-converting enzyme inhibitor; ARB, angiotensin receptor blockers; ARNI, angiotensin receptor–neprilysin inhibitor; HF, heart failure; SD, standard deviation.

## Supplementary Table S5 STROBE checklist for the reporting of observational studies

|  | Item | Recommendation | Page number(s) |
| --- | --- | --- | --- |
| **Title and abstract** | 1 | (*a*) Indicate the study’s design with a commonly used term in the title or the abstract | 1 |
|  |  | (*b*) Provide in the abstract an informative and balanced summary of what was done and what was found | 2–3 |
| Introduction | | |  |
| Background/rationale | 2 | Explain the scientific background and rationale for the investigation being reported | 5–7 |
| Objectives | 3 | State specific objectives, including any prespecified hypotheses | 6–7 |
| Methods | | |  |
| Study design | 4 | Present key elements of study design early in the paper | 7 |
| Setting | 5 | Describe the setting, locations, and relevant dates, including periods of recruitment, exposure, follow-up, and data collection | 7–9 |
| Participants | 6 | (*a*) Give the eligibility criteria, and the sources and methods of selection of participants. Describe methods of follow-up | 7–9 |
|  |  | (*b*) For matched studies, give matching criteria and number of exposed and unexposed | 8–9 |
| Variables | 7 | Clearly define all outcomes, exposures, predictors, potential confounders, and effect modifiers. Give diagnostic criteria, if applicable | 8–9 |
| Data sources/ measurement | 8* | For each variable of interest, give sources of data and details of methods of assessment (measurement). Describe comparability of assessment methods if there is more than one group | 7–10 |
| Bias | 9 | Describe any efforts to address potential sources of bias | 7–10 |
| Study size | 10 | Explain how the study size was arrived at | 8–9 |
| Quantitative variables | 11 | Explain how quantitative variables were handled in the analyses. If applicable, describe which groupings were chosen and why | 8–9 |
| Statistical methods | 12 | (*a*) Describe all statistical methods, including those used to control for confounding | 10 |
|  |  | (*b*) Describe any methods used to examine subgroups and interactions | 10 |
|  |  | (*c*) Explain how missing data were addressed | 8–9 |
|  |  | (*d*) If applicable, explain how loss to follow-up was addressed | N/A |
|  |  | (*e*) Describe any sensitivity analyses | 9 |
| Results | | |  |
| Participants | 13^a^ | (a) Report numbers of individuals at each stage of study—e.g. numbers potentially eligible, examined for eligibility, confirmed eligible, included in the study, completing follow-up, and analysed | 11  Figure 1  Supplementary figure S1 |
|  |  | (b) Give reasons for non-participation at each stage | 11  Figure 1  Supplementary figure S1 |
|  |  | (c) Consider use of a flow diagram | Figure 1  Supplementary figure S1 |
| Descriptive data | 14^a^ | (a) Give characteristics of study participants (e.g. demographic, clinical, social) and information on exposures and potential confounders | 11–13 |
|  |  | (b) Indicate number of participants with missing data for each variable of interest | Table 1 |
|  |  | (c) Summarize follow-up time (e.g. average and total amount) | 12 |
| Outcome data | 15^a^ | Report numbers of outcome events or summary measures over time | 11–13 |
| Main results | 16 | (*a*) Give unadjusted estimates and, if applicable, confounder-adjusted estimates and their precision (e.g. 95% confidence interval). Make clear which confounders were adjusted for and why they were included | 13–15  Table 2 |
|  |  | (*b*) Report category boundaries when continuous variables were categorized | 13–15  Table 2 |
|  |  | (*c*) If relevant, consider translating estimates of relative risk into absolute risk for a meaningful time period | 13–15  Table 2 |
| Other analyses | 17 | Report other analyses done—e.g. analyses of subgroups and interactions, and sensitivity analyses | 14, 15 |
| Discussion | | |  |
| Key results | 18 | Summarize key results with reference to study objectives | 15–17 |
| Limitations | 19 | Discuss limitations of the study, taking into account sources of potential bias or imprecision. Discuss both direction and magnitude of any potential bias | 21 |
| Interpretation | 20 | Give a cautious overall interpretation of results considering objectives, limitations, multiplicity of analyses, results from similar studies, and other relevant evidence | 18–20 |
| Generalizability | 21 | Discuss the generalizability (external validity) of the study results | 18–20 |
| Other information | | |  |
| Funding | 22 | Give the source of funding and the role of the funders for the present study and, if applicable, for the original study on which the present article is based | 22 |

^a^Give information separately for exposed and unexposed groups.

Source: <https://www.strobe-statement.org/index.php?id=available-checklists>

## Supplementary Figure S1 Cumulative incidence estimates with 95% confidence intervals for all-cause mortality for ARNI versus ACEi/ARB groups matched using propensity score matching including clinical variables: (A) 1:2 ratio, (B) 1:3 ratio. ACEi, angiotensin-converting enzyme inhibitor; ARB, angiotensin receptor blockers; ARNI, angiotensin receptor–neprilysin inhibitor.


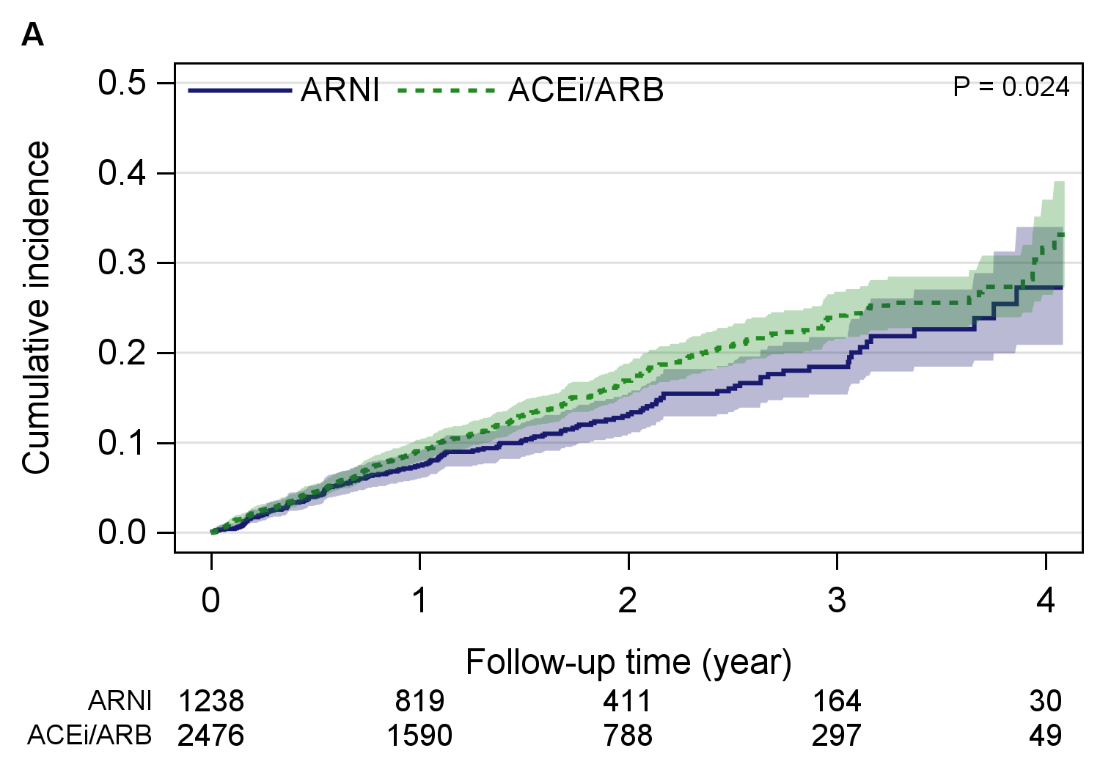

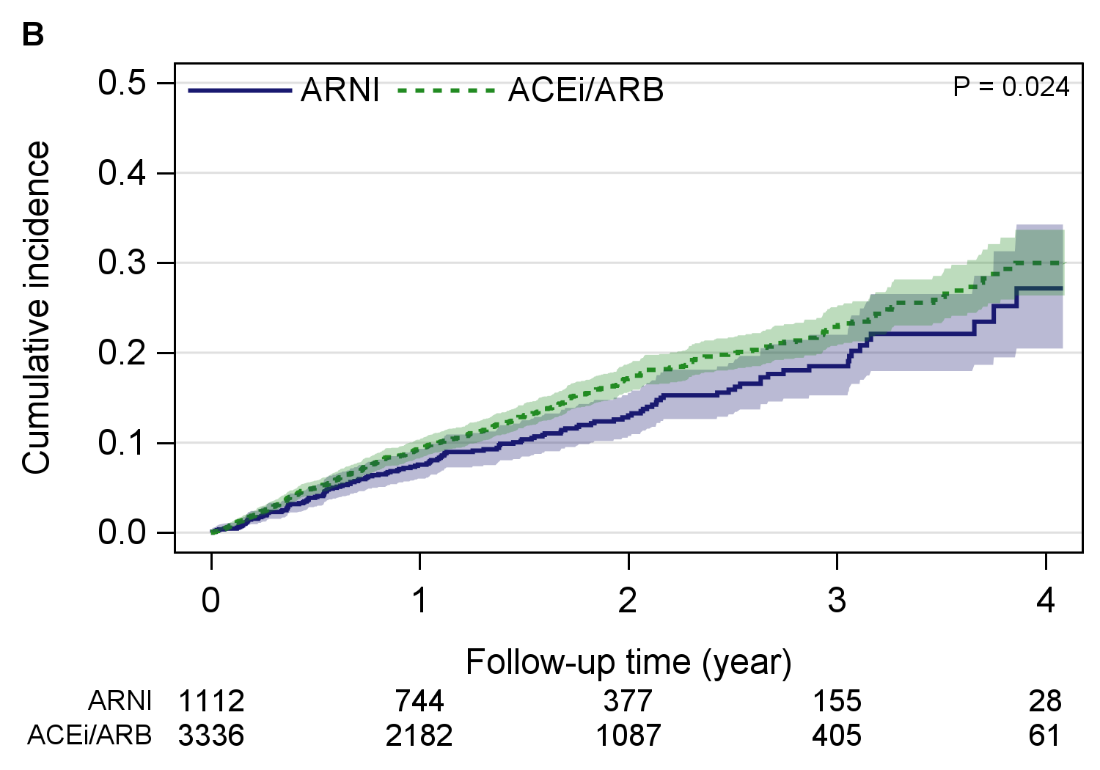


## **Supplementary Figure S2** Cumulative incidence estimates with 95% confidence intervals for cardiovascular-related mortality (handling other death as competing risk) for ARNI versus ACEi/ARB groups matched 1:1 ratio using propensity score matching including clinical variables (A) 1:2 ratio, (B) 1:3 ratio. ACEi, angiotensin-converting enzyme inhibitor; ARB, angiotensin receptor blockers; ARNI, angiotensin receptor–neprilysin inhibitor.


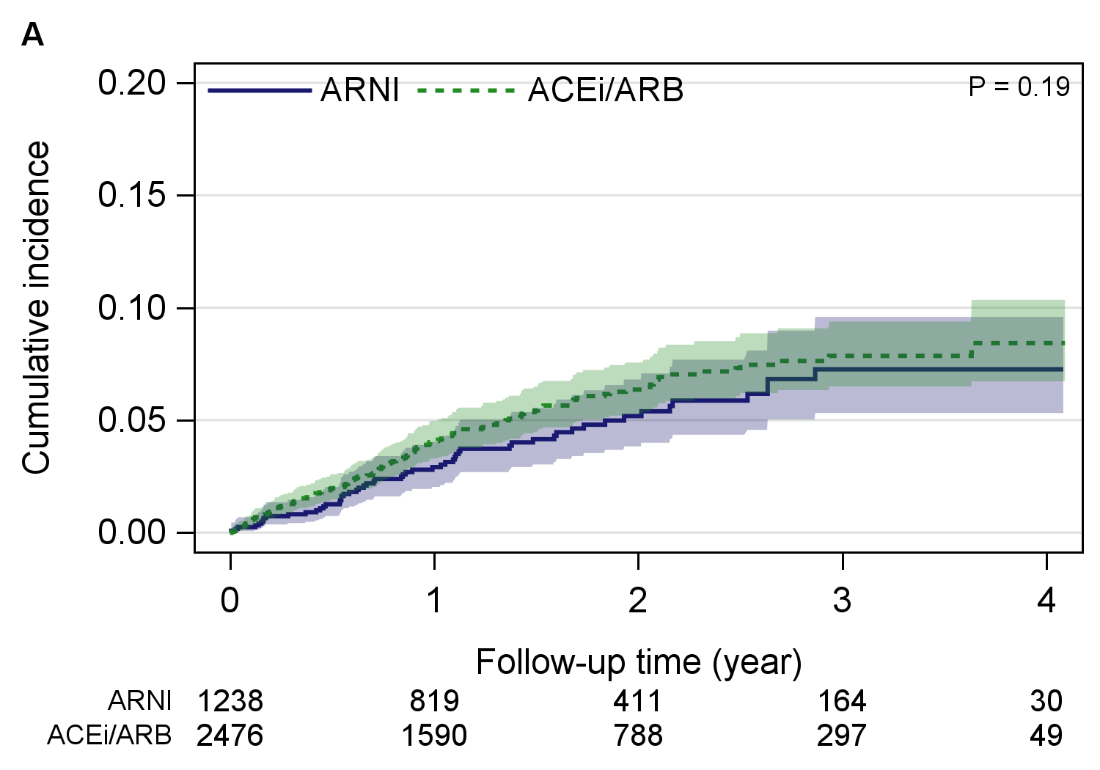

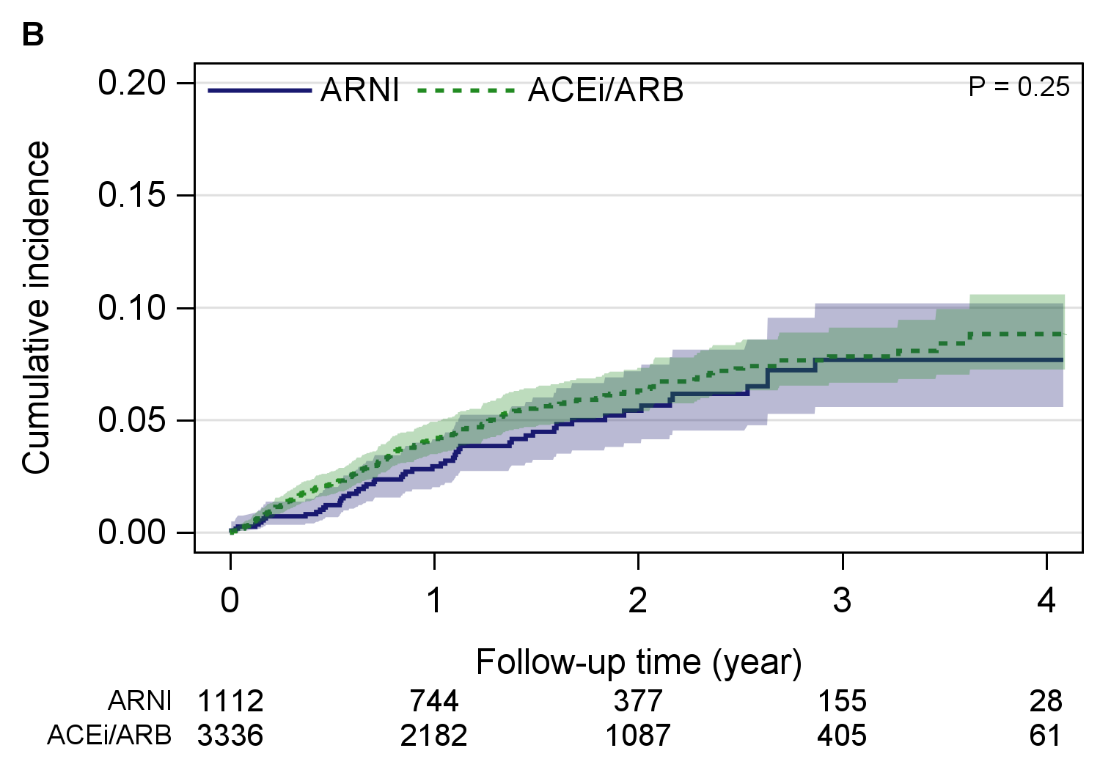


## Supplementary Figure S3 Flow diagram detailing the selection of patients used in simple exact matching without clinical variables (sensitivity analysis).

^
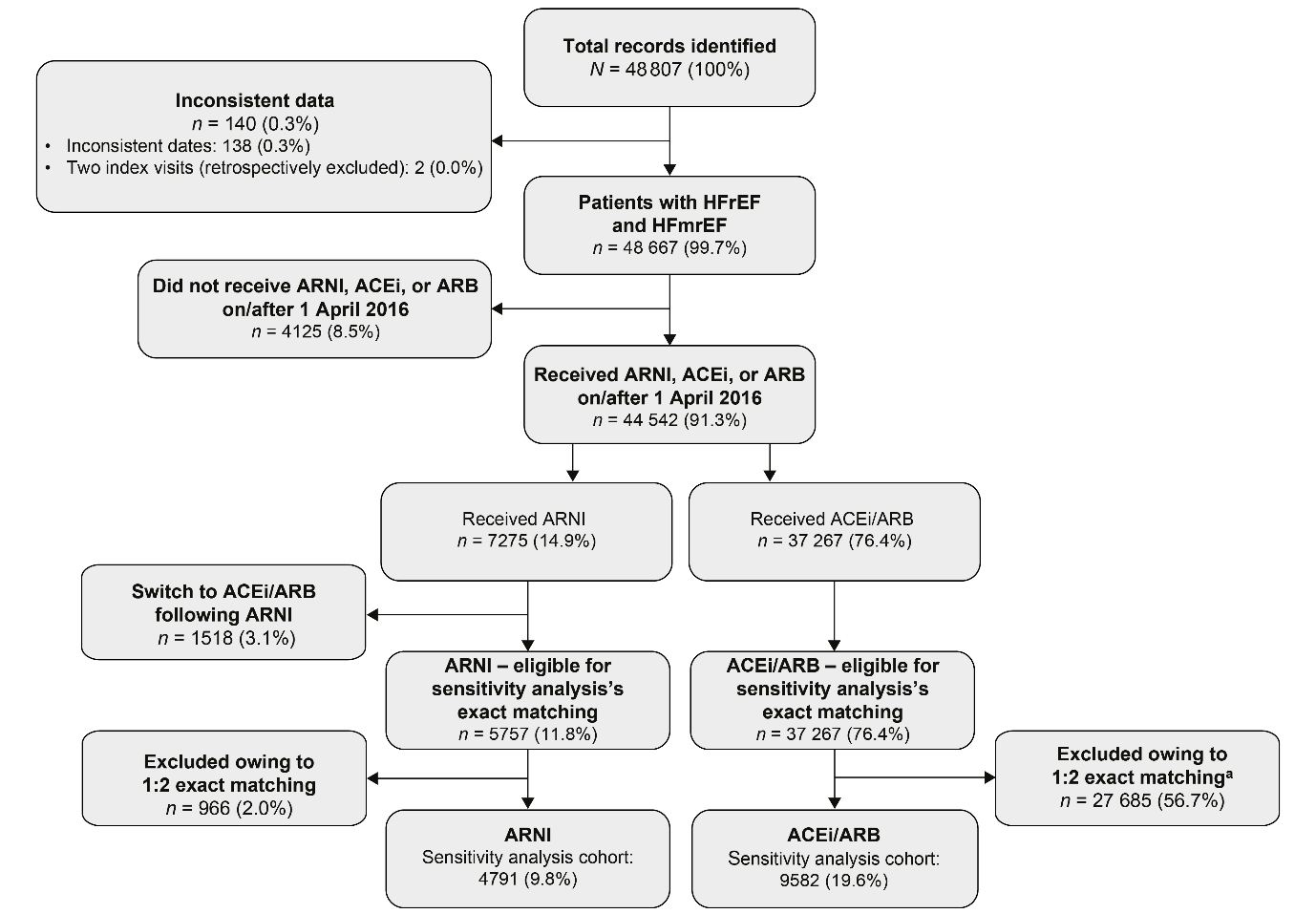
^

ACEi, angiotensin-converting enzyme inhibitor; ARB, angiotensin receptor blockers; ARNI, angiotensin receptor–neprilysin inhibitor; HFrEF, heart failure with reduced ejection fraction; LVEF, left ventricular ejection fraction.
 ^a^Patients were excluded if they were not alive or not using medication on the defined starting date for the analysis.

## Supplementary Figure S4 Cumulative incidence estimates with 95% confidence intervals for all-cause and cardiovascular-related mortality (handling other death as competing risk) for ARNI versus ACEi/ARB groups matched using 1:2 exact matching without clinical variables: (A) all-cause mortality, (B) cardiovascular-related mortality. ACEi, angiotensin-converting enzyme inhibitor; ARB, angiotensin receptor blockers; ARNI, angiotensin receptor–neprilysin inhibitor.


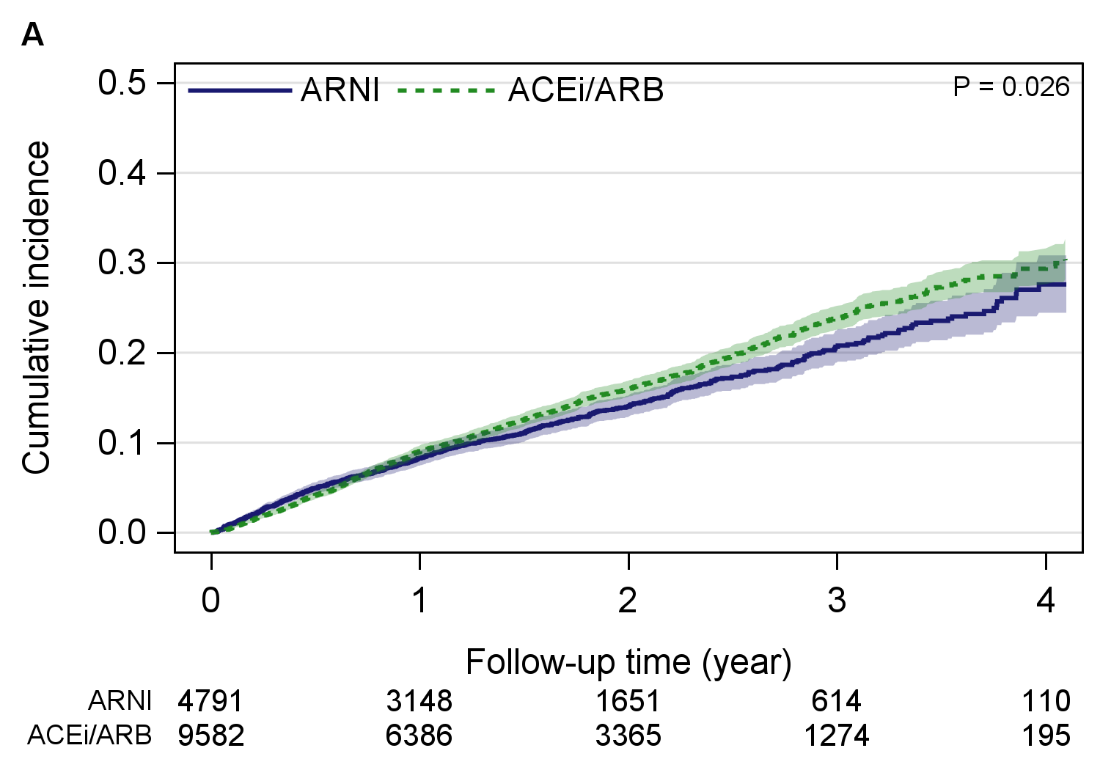

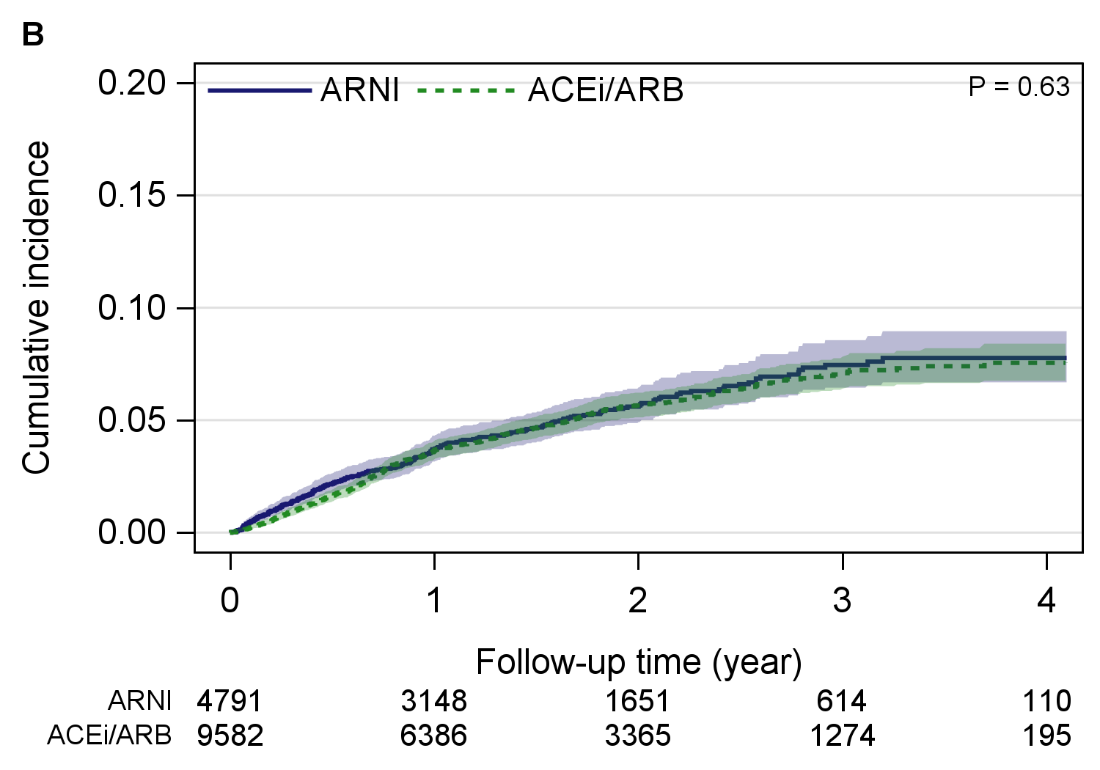

Supplement: Supplementary file 1 — Supplementary file1 (DOCX 673 kb) [file 392_2022_2124_MOESM1_ESM.docx]
